# Divergent Hantavirus in Somali Shrews (*Crocidura somalica*) in the Semi-Arid North Rift, Kenya

**DOI:** 10.3390/pathogens12050685

**Published:** 2023-05-07

**Authors:** Dorcus C. A. Omoga, David P. Tchouassi, Marietjie Venter, Edwin O. Ogola, Gilbert Rotich, Joseph N. Muthoni, Dickens O. Ondifu, Baldwyn Torto, Sandra Junglen, Rosemary Sang

**Affiliations:** 1 International Centre of Insect Physiology and Ecology, Nairobi P.O. Box 30772-00100, Kenya; 2 Zoonotic arbo and Respiratory Virus Research Program, Centre for Viral Zoonoses, Department of Medical Virology, Faculty of Health, University of Pretoria, Gezina 0031, South Africa; 3 Institute of Virology, Charité Universitätsmedizin Berlin, Corporate Member of Free University Berlin, Humboldt-University Berlin, and Berlin Institute of Health, 10117 Berlin, Germany

**Keywords:** hantavirus, shrews, phylogenetics, *Crocidura somalica*, Kenya

## Abstract

Hantaviruses are zoonotic rodent-borne viruses that are known to infect humans and cause various symptoms of disease, including hemorrhagic fever with renal and cardiopulmonary syndromes. They have a segmented single-stranded, enveloped, negative-sense RNA genome and are widely distributed. This study aimed to investigate the circulation of rodent-borne hantaviruses in peridomestic rodents and shrews in two semi-arid ecologies within the Kenyan Rift Valley. The small mammals were trapped using baited folding Sherman traps set within and around houses, then they were sedated and euthanatized through cervical dislocation before collecting blood and tissue samples (liver, kidney, spleen, and lungs). Tissue samples were screened with pan-hantavirus PCR primers, targeting the large genome segment (L) encoding the RNA-dependent RNA polymerase (RdRp). Eleven of the small mammals captured were shrews (11/489, 2.5%) and 478 (97.5%) were rodents. A cytochrome b gene-based genetic assay for shrew identification confirmed the eleven shrews sampled to be *Crocidura somalica.* Hantavirus RNA was detected in three (3/11, 27%) shrews from Baringo County. The sequences showed 93–97% nucleotide and 96–99% amino acid identities among each other, as well as 74–76% nucleotide and 79–83% amino acid identities to other shrew-borne hantaviruses, such as Tanganya virus (TNGV). The detected viruses formed a monophyletic clade with shrew-borne hantaviruses from other parts of Africa. To our knowledge, this constitutes the first report published on the circulation of hantaviruses in shrews in Kenya.

## 1. Introduction

Hantaviruses are single-stranded, enveloped, tripartite, negative-sense RNA viruses belonging to the order of *Bunyavirales* in the family *Hantaviridae*, subfamily *Mammantavirinae,* and can be classified into four genera: *Orthohantavirus, Loanvirus, Mobatvirus*, and *Thottimvirus* [1,2]. They have been reported in diverse small mammals, including rodents, shrews, moles, and bats [3,4,5,6,7,8,9]. However, recently reported and previously unknown hantaviruses have been found in Africa and Asia, mainly in shrews and moles, as well as in bats [8,10,11]. Hantaviruses typically cause chronic asymptomatic infection in rodents and severe diseases in humans, such as hemorrhagic fever with renal syndrome (HFRS) and hemorrhagic cardiopulmonary syndrome (HCPS), with a case-fatality rate in humans of up to 40% [10,12,13,14]. Yearly, approximately 150,000 to 200,000 cases of hantavirus disease occur [15]. These cases have been mostly reported in Asia, wherein 70–90% represent HFRS cases in China; nevertheless, there has been an increase in the number of cases reported in America and Europe [15]. By the year 2020, in the United States of America (USA), there had been an estimated 833 laboratory-confirmed cases of hantavirus infection [16].

HFRS is caused by “Old World” hantaviruses, such as Hantaan virus (HTNV), Puumula virus (PUUV), Dobrava virus (DOBV), and Seoul virus (SEOV), among others, and is common in Europe and Asia. On the other hand, HCPS is caused by “New World” hantaviruses such as Bayou virus (BAYV), Black Creek Canal virus (BCCV), Sin Nombre virus (SNV), and Andes virus (ANDV) in the Americas [17]. Comparing the two-hantavirus infections in terms of disease prognosis, HFRS has a high morbidity rate characterized by a mild-to-moderate infection that presents as fever, headaches, inflammation or redness of the eyes, rashes, and gastrointestinal symptoms, and may develop into acute kidney failure and hypotension; however, the fatality rate is low (1–5%) depending upon which virus is causing the disease [15,17,18,19]. In contrast, HCPS has a high case-fatality rate and up to 60% mortality has been reported in some outbreaks. Symptoms include fatigue, fever, muscle aches, headaches, dizziness, chills, and gastrointestinal symptoms and may progress to coughing, shortness of breath, and chest congestion. The high fatality rate of HCPS is attributed to the attendant respiratory and pulmonary complications [15,17,20,21].

Currently, there is no specific treatment, cure, or vaccine for hantavirus infection; hence, early diagnosis, proper management, and adequate support can increase survival in approximately 70–80% of confirmed cases [16,19,21].

Hantaviruses are transmitted to humans through inhalation of the aerosolized virus, the consumption of contaminated foods, and contact with infected urine, feces, and saliva, and less frequently by a bite from an infected rodent host [22,23]. Exceptionally, the only reported human-to-human hantavirus transmission through body fluids and direct contact has been reported for ANDV in some studies in Chile and Argentina [24,25,26]. However, a recent review by Toledo et al. in 2021 illustrated some limitations to the reported evidence of human-to-human transmission since the reports are only limited to ANDV and do not include any other hantavirus strains, as well as the lack of the same evidence in Europe and Asia and other counties in the Americas, where the virus is also present [27]. Another contentious issue is the fact that this mode of transmission has only been reported in some studies but not others in both Chile and Argentina [28,29,30]. Hence, there is a need for advanced, well-designed studies to provide sufficient evidence as to human-to-human transmission to inform public health authorities.

Among rodents, hantaviruses are transmitted either through direct contact or indirectly through the inhalation of aerosolized viral particles from rodent excreta. Therefore, people in frequent contact with rodents and rodent-infested areas are at a high risk of being infected [31,32].

Temperate weather conditions are favorable for small mammal populations, while harsh weather conditions such as droughts might drive these animals indoors in search of food, thus increasing both their contact with humans and the possibility of infection. In addition, the availability of food influences the size of the rodent population; for instance, they are notably abundant during the harvesting period due to widely available food sources, which enhances reproduction. As harvested foods are mainly stored inside houses and in granaries/stores adjacent to houses, the likelihood of human interaction with rodents is higher in these instances. Hence, the risk of hantavirus infection and disease incidences are positively correlated with rodent population size, infestation, and abundance [33].

Hantaviruses are distributed worldwide and are known to occur in the Americas, Asia, and Europe, but there is little knowledge on hantavirus distribution in Africa, especially in sub-Saharan Africa. Outside Africa, shrew-borne hantaviruses have been reported in various shrew species, including the Seewis virus (SWSV) from the Eurasian common shrew (*Sorex araneus*) in Switzerland [34] and the Boginia virus (BOGV) from the Eurasian water shrew (*Neomys fodiens*) in Poland [35], among others.

In Africa, the first indigenous hantavirus, Sangassou virus (SANGV) was isolated from the African wood mouse (*Hylomyscus simus*) in Guinea [3]. Another shrew-borne hantavirus, Tanganya virus (TNGV), was later detected in *Crocidura theresae* in the same country [36]. Other shrew-borne hantaviruses from Africa include the Azagny virus (AZGV), found in a West African pygmy shrew (*Crocidura obscurior*) from the Côte d’Ivoire [9], Kilimanjaro virus (KMJV), and Uluguru virus (ULUV), detected in the Kilimanjaro mouse shrew *(Myosorex zinki*) and the Geata mouse shrew (*Myosorex geata*) in Tanzania [37], respectively.

Furthermore, sero-epidemiological studies in Africa have revealed the presence of antibodies against hantaviruses in humans, including febrile patients with a fever of unknown origin (FUO); for example, a 4.4% seroprevalence rate against SANGV was found in Guinea [38]. Additionally, SANGV IgM antibodies were detected in a serum sample collected from a child presenting with fever and other HFRS-like symptoms, such as nausea, dyspnea, muscle pain, oliguria, vomiting, and a swollen face and legs [38].

In Kenya, hantavirus RNA has so far only been detected in an African wood mouse (*Hylomyscus endorobae*) [39]. A recently conducted study among febrile patients from the Kibera informal settlement in Nairobi reported reactivity in 8.1% of the tested sera with Puumala orthohantavirus and Dobrava–Belgrade orthohantavirus in immunofluorescence assays, suggesting that hantaviruses are circulating in Kenya and could be contributing to febrile illnesses [40].

In this research, we sought to gain further insight into the presence of hantavirus in shrews and rodents in Kenya.

## 2. Materials and Methods

### 2.1. Study Sites

**Figure 1:**
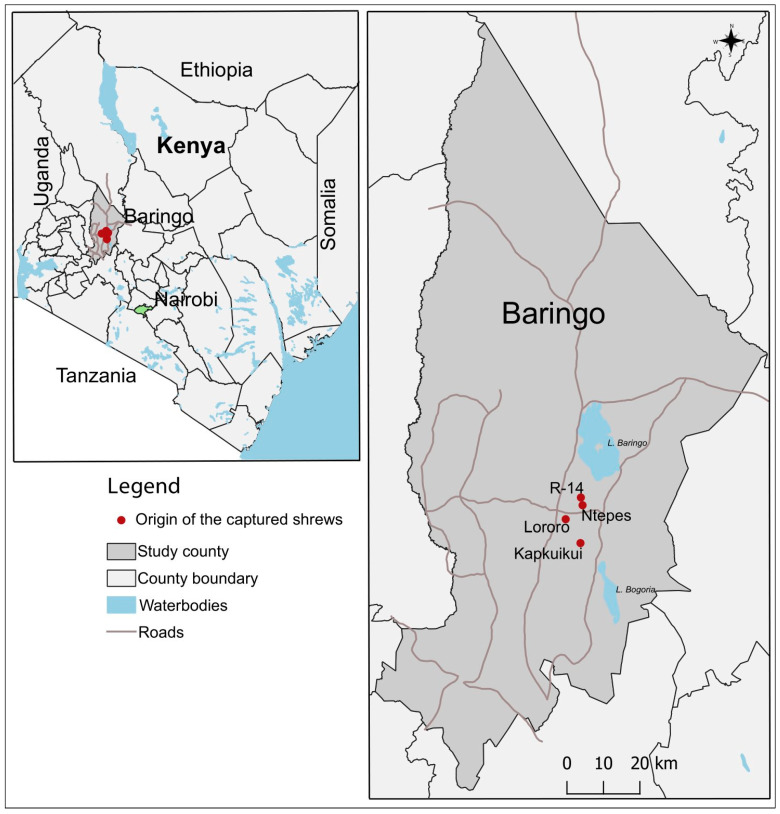
A map of Kenya showing the two study counties (Baringo and Kajiado); sites in Baringo county where the shrews (*Crocidura somalica*) were captured are represented by red spots. The maps were created in the open-source GIS software, QGIS 3.22 using GPS coordinates and shape files derived from Natural Earth (http://www.naturalearthdata.com/, a free GIS data source, accessed on 20 April 2023) and Africa Open data (https://africaopendata.org/dataset/kenya-counties-shapefile, licensed by the Creative Commons, accessed on 20 April 2023) [42].

The sampling of peridomestic rodents and shrews was performed in diverse ecologies along the Rift Valley in Kenya (Figure 1). These were Marigat (0.4695° N, 35.9833° E) in Baringo County and Nguruman (1.7617° S, 36.0255° E) in Kajiado County, both semi-arid areas with diverse populations and high human/livestock/wildlife interactions [41]. The two ecologies are both inhabited by semi-nomadic agro-pastoralist communities, who keep livestock and grow maize, millet, and fruits in irrigated farmlands. The availability of these foods supports rodent populations.

### 2.2. Peridomestic Rodents and Shrews Sampling

Rodents were trapped using LFAHD Folding Live Capture Rodent/Rat/Mouse Traps (3 × 3.5 × 9” (7.62 × 8.89 × 2.86 cm)) and SFA Small Folding Live Capture Rodent/Vole/Shrew/Mouse Traps (2 × 2.5 × 6.5” (5.08 × 6.35 × 16.51 cm)) (HB Sherman, Tallahassee, FL, USA). Sampling was performed according to the National Museum of Kenya (NMK) guidelines for trapping and sampling rodents in Kenya [43]. Trapping was conducted for a period of two weeks twice a year during or immediately after harvesting when the rodent activity was presumed to be high. First, the traps were baited with a mixture of locally available peanut butter and white oats, then set inside dwelling places (houses, shops, and stores) and the surrounding area (fences and adjacent farms). Fifty traps were set per night, with a varied number of traps set per site depending on the observed rodent activity and the confirmation of rodent presence from the residents. The baited traps were set at dusk, checked, and collected every morning for two consecutive days per site. All the collected traps were transported to the field laboratory, where the captured rodents were placed in a sampling bag, then all the traps were emptied and cleaned. During trap resetting at the same sampling site, if any interference was noticed upon inspection, the trap was then moved to a different location within the site after rebaiting.

The trapped specimen that was placed in the handling bag was first euthanatized by cervical dislocation and then identified to the genus level, based on morphological and geographical criteria chosen according to *The Kingdom Guide to African Mammals and East African Mammals* and further by molecular analyses [4,43,44]. Parameters such as species, sex, age, length, and weight were recorded from trapped individuals. Immediately after ensuring that the mice were completely euthanized, first, blood samples were collected from the heart through cardiac puncture and thoracotomy, followed by the collection of other tissue samples (from the kidney, spleen, lungs, heart, and liver). The collected samples were appropriately labeled and then stored in liquid nitrogen, before transportation to the Martin Lüscher Emerging Infectious Diseases (ML-EID) Laboratory at the International Centre of Insect Physiology and Ecology (*icipe*) for immediate testing and/or storage at −80 °C until further screening.

### 2.3. RNA Extraction, cDNA Synthesis, and PCR Detection of Hantaviruses

Approximately 200 mg of pooled tissue sample (50 mg of each lung, spleen, kidney, and liver) from an individual rodent was homogenized in 500 μL PBS in a Geno/Grinder 2010 (ATS Scientific Inc, Burlington, Canada). The homogenized samples were then separated by centrifugation at 3000 rpm for 5 min in an Eppendorf™ Microcentrifuge 5430 benchtop centrifuge (Eppendorf SE, Hamburg, Germany). RNA was extracted from 140 µL of the supernatant of the homogenized rodent tissues using the QIAamp Viral RNA Minikit (QIAGEN, Hilden, Germany), according to the manufacturer’s protocol. A volume of 50 μL of RNA was obtained and used as a template for cDNA synthesis with Invitrogen SuperScript™ III Reverse Transcriptase, according to the manufacturer’s instructions. The cDNA was stored at −80°C until further use.

The samples were screened for hantavirus RNA by an optimized assay targeting partial polymerase (L) gene sequences of the hantavirus genomes, using the primers Han-L-F1:5’-ATGTAYGTBAGTGCWGATGC-3’, Han-L-R1:5’-AACCADTCWGTYCCRTCATC-3’, Han-L-F2:5’-TGCWGATGCHACIAARTGGTC-3’, and Han-L-R2:5’-GCRTCRTCWGARTGRTGDGCAA-3’ [3]. The reaction volume (25 µL) comprised 15.65 µL PCR water, 2.50 µL 10×buffer, 1.25 µL Mg (50 mM), 0.50 µL dNTPs (10 mM), 1.5 µL of 10 µM forward and reverse primers, 0.10 µL Platinum-Taq polymerase, and 2.0 µL of the template (cDNA).

The following conditions were used for the PCR reaction: 98 °C for 30 s, 35 cycles of 98 °C for 10 s, 50 °C for 30 s, and 72 °C for 30 s, with a final step of 72 °C for 7 min and 4 °C for infinity.

The PCR products were electrophoresed in 2% agarose gel stained with ethidium bromide (Sigma-Aldrich Chemie GmbH, Taufkirchen, Germany ) and positive samples were purified using ExoSAP-IT™ PCR Product Clean-up Reagent (Thermo Fisher Scientific Baltics UAB, Vilnius, Lithuania) according to the manufacturer’s instructions, then the samples were sequenced in both directions (see Section 2.4).

In an attempt to obtain orthohantavirus whole-genome sequences via next-generation sequencing (NGS), RNA was extracted from hantavirus-positive tissue samples and libraries prepared using the KAPA HyperPlus kit (Roche, Penzberg, Germany) and then sequenced by the Illumina MiSeq HTS platform (Illumina, Inc., San Diego, USA ) [45,46].

### 2.4. Sanger Sequencing and Phylogenetic Analysis

The Sanger sequencing services were outsourced from Macrogen, Europe BV (Macrogen, Amsterdam, The Netherlands) for both forward and reverse strands, using the Han-L-F2: 5’-TGCWGATGCHACIAARTGGTC-3’; and Han-L-R2: 5’-GCRTCRTCWGARTGRTGDGCAA-3’ primers. The sequences were cleaned using the Geneious Prime software (http://www.geneious.com, accessed on 16 April 2023) and then queried with the GenBank-NCBI database [47,48] using the basic local alignment search tool (BLAST) [49]. Related sequences were downloaded from the GenBank-NCBI database and multiple sequence alignment was performed by MAFFT [50], then phylogenetic analysis with PhyML [51] was conducted, using the general time-reversible (GTR) model and applying 1000 bootstrap replicates. All analyses were performed with Geneious Prime (http://www.geneious.com, accessed on 16 April 2023), using the default parameters.

### 2.5. Rodent and Shrew Identification

Species identification of rodents and shrews was performed by extracting DNA from the previously prepared pooled tissue samples (see Section 2.3), using the Qiagen DNeasy Blood & Tissue extraction kit (QIAGEN, Hilden, Germany) according to the manufacturer’s instructions. The shrew barcoding was performed using the forward primer, sorcytb365F (5’-CAGTAATAGCCACTGCCTTTATAGG-3’), and the reverse primer, sorcytb969R (5’-CATTGGCTGAATGGGCGGAATATTAT-3’), which target a 500 bp region of the cytochrome b (*cyt b*) gene [4]. For rodent identification with the cytochrome c oxidase 1 gene (*COI*) barcode region, the specific primers BatL5310: 5′-CCTACTCRGCCATTTTACCTATG-3′ and R6036R: 5′- ACTTCTGGGTGTCCAAAGAATCA-3′ were used [52,53,54]. The following conditions were used for the PCR reaction: 95 °C for 3 min; 35 cycles of 95 °C for 10 s, 55 °C for 30 s, 72 °C for 1 min, and then 72 °C for 7 min followed by a hold at 4 °C.

Gel electrophoresis of the PCR products was performed in 1.5% agarose gel stained with ethidium bromide (Sigma-Aldrich Chemie GmbH, Taufkirchen, Germany), then the positive samples were purified for sequencing with ExoSAP-IT™ PCR Product Clean-up Reagent (Thermo Fisher Scientific Baltics UAB, Vilnius, Lithuania), according to the manufacturer’s instructions, and then sequenced in both directions by Macrogen, Europe BV (Macrogen, Amsterdam, The Netherlands). Phylogenetic analysis was performed using Geneious Prime (http://www.geneious.com, accessed on 16 April 2023), based on the nucleotide sequences of the L segment. Related sequences were downloaded from the GenBank-NCBI database and multiple sequence alignment was performed by MAFFT [50]. A phylogenetic tree was constructed with PhyML [51] and the GTR substitution model, applying 1000 bootstrap replicates.

## 3. Results

### 3.1. Rodent Distribution

A total of 489 peridomestic small mammals (rodents and shrews) were captured in Nguruman (63.8%, 312/489) and Marigat (36.2%, 177/489). Eleven of these were shrews (11/489, 2.5%), while the other rodents (478/489, 97.5%) belonged to at least 14 different species (Table 1). Of the 312 small mammals in total that were captured in Nguruman, 48% were captured indoors and 52% were captured outdoors. The percentage of males captured was higher (55%) compared to females (45%) (Table 1).

In contrast, in Marigat, out of the 177 small mammals captured, 80% were captured indoors and 20% outdoors, and the number of females was higher (58%) than of males (42%) (Table 1). The most common species were the *Mastomys* spp. and the least common, *Oenomys* spp., *Graphiurus* spp., *Paraxerus* spp., and *Gerbillus* spp.

All 11 shrews were captured in Marigat, while none were captured in Nguruman (Figure 1). Of these 11, 10 were captured outdoors (90.9%, 10/11), and one indoors (9.1%, 1/11) in Lororo, Mchongoi village.

### 3.2. Hantavirus Detection, Characterization, and Phylogenetic Analysis

**Figure 2:**
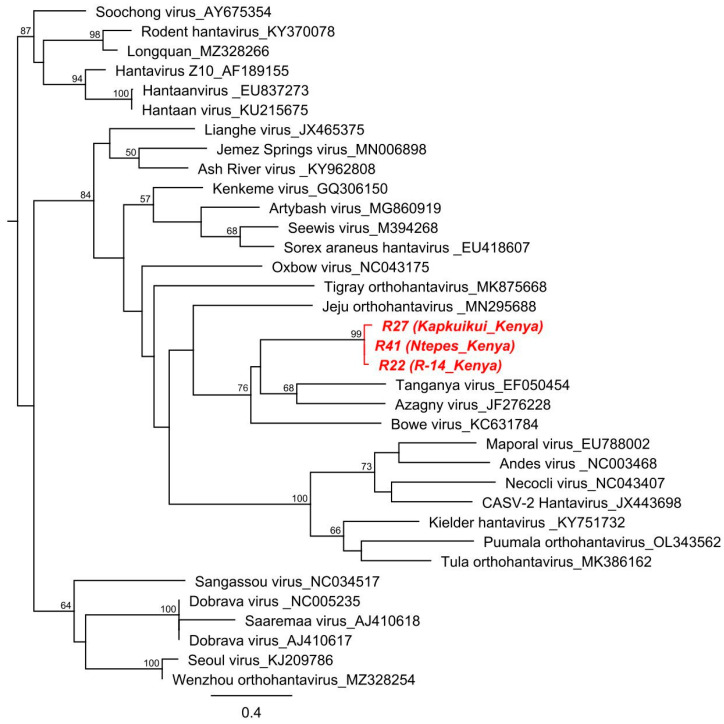
Phylogenetic relationship of hantavirus in this study. A maximum-likelihood (ML) phylogenetic analysis, based on 385-nucleotide fragments of the RNA-dependent RNA polymerase gene of hantaviruses detected in this study (R22, R27, and R41) and other members of the family *Hantaviridae*, genus *Orthohantavirus*, was performed using MAFFT, PhyML v. 2.2.4, with a GTR substitution model and employing 1000 bootstrap replicates. Sequences identified in this study are shown in red (GenBank accession numbers: OQ709085–OQ709088).

We detected hantavirus RNA in three out of the eleven shrews (3/11, 27%) captured in Marigat. The amplicon sequences of 385 nucleotides showed 93–97% nucleotide and 96–99% amino acid identities among each other and low levels (74–76%) of nucleotide and 79–83% amino acid identities to other shrew-borne hantaviruses, such as TNGV, AZGV, and SWSV, across the partial polymerase (RdRp) gene region that was characterized in this study (Figure 2). No hantavirus RNA was detected in any of the rodent species.

Attempts to obtain more genome information on the hantavirus-positive shrew samples via next-generation sequencing were not successful.

### 3.3. Barcoding Identification of Shrews

**Figure 3:**
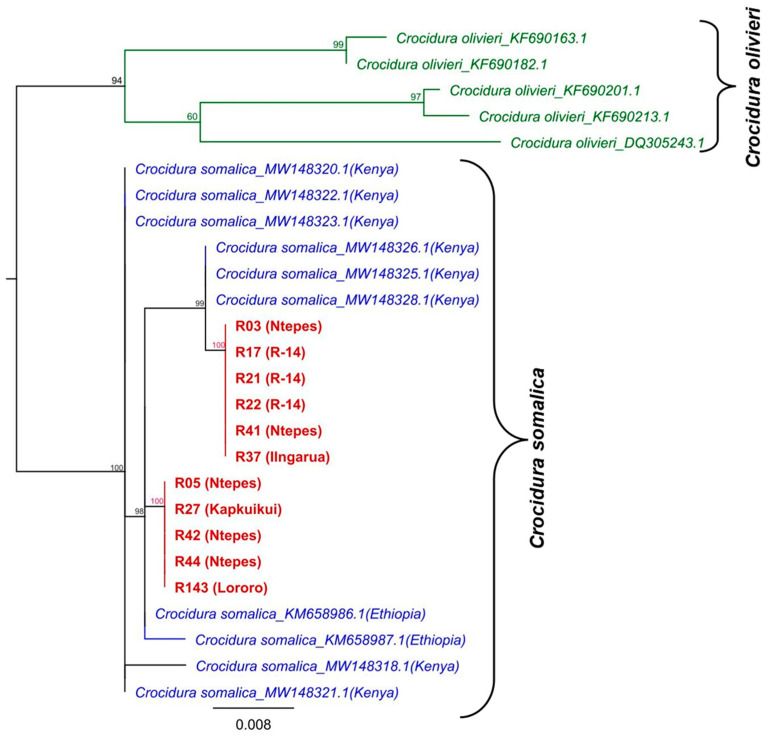
Phylogenetic relationship of the *CytB* gene sequences detected in this study. ML phylogenetic analysis, based on the 500 bp partial sequence of the *CytB* gene and other sequences from *Crocidura somalica* and *Crocidura olivieri* (outgroup) obtained from the GenBank, were aligned using MAFFT and the tree inferred using PhyML v. 2.2.4 with GTR substitution models employing 1000 bootstrap replicates. All the sequences generated from the shrews in this study are shown in red (GenBank and the accession numbers: OQ709089–OQ709099). Taxon names include the species designation, Genbank accession number, and country (in brackets).

Sequence analysis of the cytochrome b (*CytB*) gene of the eleven shrew samples confirmed the species identity of the captured shrews to be the Somali shrew, *Crocidura somalica,* based on the 99–100% nucleotide sequence identity to shrews previously captured in the same area as part of a prior study [55]. The obtained sequences had a 99–100% nucleotide identity among each other. Phylogenetic analysis revealed the formation of monophylogenetic clustering, not only with shrews obtained from Kenya but also with two sister clades (Figure 3).

## 4. Discussion

Herein, we report the detection of three hantavirus sequences in Somali shrews (*Crocidura somalica*) and the distribution patterns of peridomestic small mammals in two ecosystems in the Rift Valley of Kenya. To our knowledge, this is the first reported detection of a shrew-borne hantavirus in Kenya.

Peridomestic small mammals are in close contact with humans due to shared habitats, thereby presenting a risk of transmitting zoonotic diseases. Human activities, such as farming and building shelters, are known to interfere with the natural habitat of these animals; however, this may also create the perfect environment for rodent infestation, frequent contact, and an increased chance of disease spillover, as in the case of the house mouse, which has adapted to inhabiting human settlements. Farming activities present a conducive environment for habitation and the storage of farmed products, which provide food sources and attract rodents. Consequently, surveillance for rodent-borne zoonotic pathogens is important in such an environment.

Hantaviruses are widely distributed; the greatest number of infections has been reported in Asia and the fewest in Africa. This might be caused by enhanced surveillance and better diagnosis and reporting systems in Asia, compared to those in Africa. Other than rodent- and bat-borne hantaviruses, shrew-borne hantaviruses have been reported in Asia and Europe, and occasionally in Africa as well, e.g., detections of TGNV and AZGV, among others [9,36].

The sequences from this study that were detected in the Somali shrew (*Crocidura somalica*) were most closely related to other shrew-borne hantaviruses in terms of phylogenetic analysis. The three virus sequences formed a monophyletic lineage (99% bootstrap support) and were clustered within a clade containing TGNV, AZGV, Bowe virus (BOWV), and Jeju virus (JJUV). The genetic distance, together with confirmation of monophyly, indicate that the virus strains circulating in Kenya represent a different species that is distinct from strains reported in other regions of Africa, as well as other parts of the world. Considering that the sequences were all detected in the same shrew species, the nucleotide and amino acid sequence similarities of approximately 95% and 98%, respectively, among the new virus strains indicate the possibility that these sequences were from one virus strain circulating in the same shrew species. However, further studies that include the generation of complete genome sequences and the testing of shrews from other regions are necessary to assess the genetic diversity of hantaviruses in Kenyan shrews and enable phylogenetic classification.

Species demarcation criteria for the family *Hantaviridae* are based on the amino acid distances of the glycoprotein (M segment) and nucleoprotein (S segment) genes, assessed via diversity partitioning by hierarchical clustering (DEmARC) analysis with a cut-off value of 0.1 in the pairwise evolutionary distance (PED) [2]. Herein, we present three partial L-segment sequences that showed maximum amino acid identities of 79–83% to TGNV. The high levels of genetic distance from established hantavirus species suggest the detection of at least one distinct hantavirus in Kenya [9,36]. However, as outlined above, entire genome sequences are crucial for classification. Attempts to obtain genome sequences from the virus-positive tissue samples were not successful and virus isolation could not be attempted due to the high-containment laboratory requirement for hantavirus isolation.

Knowledge of hantavirus infection in humans in Africa is scarce apart from the reports from Djibouti, the Côte d’Ivoire, the Democratic Republic of Congo, Gabon, and Nairobi, which suggest human exposure to hantaviruses [40,56,57,58]. Thus, epidemiologic studies are needed to shed light on human hantavirus infections in Africa and the potential disease burden caused by such infections.

To our knowledge, this is the first report associating *Crocidura somalica* shrew species with hantavirus. Hantaviruses have been detected in diverse shrew species, both in Africa and other parts of the world [3,9,34,35,36,59,60], and, considering the wide distribution of this species, further screening would be important to confirm the circulation of this strain of hantavirus in species from other geographical regions.

The geographical distribution of shrews in Kenya is unknown. *Crocidura somalica* was found in the same area in a previous study [55], which is an indication that the species commonly inhabits the region. Although the Somali shrew is widely distributed and is known to have adapted to a wide variety of environments, it is mainly found in arid habitats, such as dry savanna and semi-desert areas [61]. In this study, the shrews were only captured in the Marigat area (Baringo South sub-county) and not in Nguruman (Kajiado West sub-county), which provides further evidence for their distribution in dry regions. All the shrews were captured outdoors along fences and on farms except for one from Lororo, Mchongoi that was captured indoors. Ecosystem characteristics determine the abundance patterns of small mammals [62]. Comparing the two semi-arid study sites, Marigat has a more settled human population with permanent houses/structures and thus may be more conducive for certain small mammal species; for example, the common house mouse (*Mus musculus*) was mainly captured in Marigat and was not caught in Nguruman. The settlements in Nguruman often consist of temporary shelters that are only temporarily inhabited by semi-nomadic pastoralists. For example, *Mastomys natalensis,* the most prevalent rodent species captured in this study, was found in higher abundance in Nguruman, which may indicate a preference for shrubland ecosystems. However, comprehensive ecological studies are needed to identify the habitat requirements for small mammals in East Africa.

## 5. Conclusions

In this study, we report the detection of hantaviruses in Somali shrews sampled in Baringo County (Marigat), a semi-arid ecology dominated by pastoralist communities and farmers. To our knowledge, this is the first report of a shrew-borne hantavirus in Kenya. The findings generally provide the basis for further screening and characterization to improve our understanding of the geographical distribution of shrews, the strains of hantaviruses, and their probable host species. However, further studies are needed to determine the detected hantaviruses’ full genome sequences and investigate virus distribution and host range.

## Figures and Tables

**Table 1:** Distribution of small mammals from the two study sites.

Parameter	Level	Site		
		Marigat	Nguruman	Total
Sex	Female	102	140	242
	Male	75	172	247
Age	Sub-Adult	36	118	154
	Adult	141	194	335
Place of Capture	Indoor	137	150	287
	Outdoor	40	162	202
Species	*Acomys* spp.	1	48	49
	*Aethomys* spp.	1	6	7
	*Arvicanthis* spp.	0	15	15
	*Oenomys* spp.	1	0	1
	*Lemniscomys* spp.	0	2	2
	*Gerbilliscus* spp.	3	2	5
	*Grammomys* spp.	2	0	2
	*Graphiurus* spp.	0	1	1
	*Mastomys* spp.	47	217	264
	*Mus* spp.	73	0	73
	*Paraxerus* spp.	0	1	1
	*Gerbillus* spp.	0	1	1
	*Crocidura* spp.	11	0	11
	*Rattus* spp.	38	19	57

## Data Availability

The data presented in this study are available on request from the corresponding author. The data are not publicly available due to planned future studies. The partial sequences of the L segment and the cytochrome b were deposited in GenBank under the following accession numbers: OQ709085-OQ709099.

## References

[1] Kuhn J.H., Adkins S., Agwanda B.R., Al Kubrusli R., Alkhovsky S.V., Amarasinghe G.K., Avšič-Županc T., Ayllón M.A., Bahl J., Balkema-Buschmann A. (2021). 2021 Taxonomic update of phylum Negarnaviricota (Riboviria: Orthornavirae), including the large orders Bunyavirales and Mononegavirales. Arch. Virol..

[2] Laenen L., Vergote V., Calisher C.H., Klempa B., Klingström J., Kuhn J.H., Maes P. (2019). Hantaviridae: Current Classification and Future Perspectives. Viruses.

[3] Klempa B., Fichet-Calvet E., Lecompte E., Auste B., Aniskin V., Meisel H., Denys C., Koivogui L., Ter Meulen J., Krüger D.H. (2006). Hantavirus in African Wood Mouse, Guinea. Emerg. Infect. Dis..

[4] Lwande O.W., Mohamed N., Bucht G., Ahlm C., Olsson G., Evander M. (2020). Seewis Hantavirus in Common Shrew (*Sorex araneus*) in Sweden. Virol. J..

[5] Dubois A., Galan M., Cosson J.F., Gauffre B., Henttonen H., Niemimaa J., Razzauti M., Voutilainen L., Vitalis R., Guivier E. (2017). Microevolution of Bank Voles (*Myodes glareolus*) at Neutral and Immune-Related Genes during Multiannual Dynamic Cycles: Consequences for *Puumala Hantavirus* Epidemiology. Infect. Genet. Evol..

[6] Zhang Y.Z. (2014). Discovery of Hantaviruses in Bats and Insectivores and the Evolution of the Genus *Hantavirus*. Virus Res..

[7] Yashina L.N., Abramov S.A., Gutorov V.V., Dupal T.A., Krivopalov A.V., Panov V.V., Danchinova G.A., Vinogradov V.V., Luchnikova E.M., Hay J. (2010). Seewis Virus: Phylogeography of a Shrew-Borne Hantavirus in Siberia, Russia. Vector-Borne Zoonotic Dis..

[8] Arai S., Yanagihara R. (2020). Genetic Diversity and Geographic Distribution of Bat-Borne Hantaviruses. Curr. Issues Mol. Biol..

[9] Kang H.J., Kadjo B., Dubey S., Jacquet F., Yanagihara R. (2011). Molecular Evolution of Azagny Virus, a Newfound Hantavirus Harbored by the West African Pygmy Shrew (*Crocidura obscurior*) in Côte d’Ivoire. Virol. J..

[10] Lee H.W., Vaheri A., Schmaljohn C.S. (2014). Discovery of Hantaviruses and of the Hantavirus Genus: Personal and Historical Perspectives of the Presidents of the International Society of Hantaviruses. Virus Res..

[11] Guterres A., de Oliveira R.C., Fernandes J., de Lemos E.R.S., Schrago C.G. (2017). New Bunya-Like Viruses: Highlighting Their Relations. Infect. Genet. Evol..

[12] D’Souza M.H., Patel T.R. (2020). Biodefense Implications of New-World Hantaviruses. Front. Bioeng. Biotechnol..

[13] Tian H., Tie W.-F., Li H., Hu X., Xie G.-C., Du L.-Y., Guo W.-P. (2020). Orthohantaviruses Infections in Humans and Rodents in Baoji, China. PLoS Negl. Trop. Dis..

[14] Kim W.K., Cho S., Lee S.H., No J.S., Lee G.Y., Park K., Lee D., Jeong S.T., Song J.W. (2021). Genomic Epidemiology and Active Surveillance to Investigate Outbreaks of Hantaviruses. Front. Cell. Infect. Microbiol..

[15] Manigold T., Vial P. (2014). Human Hantavirus Infections: Epidemiology, Clinical Features, Pathogenesis and Immunology. Swiss Med. Wkly..

[16] de Lemos E.R.S., Fernandes J., Coelho T.A., Lumi L.O., Rosa J.A.R., Biasus L., Nunes Z.M.A., Brack D.B., de Oliveira R.C. (2022). Case Report: Hantavirus Cardiopulmonary Syndrome Diagnostic in the Face of the COVID-19 Pandemic. Am. J. Trop. Med. Hyg..

[17] Jonsson C.B., Figueiredo L.T.M., Vapalahti O. (2010). A Global Perspective on Hantavirus Ecology, Epidemiology, and Disease. Clin. Microbiol. Rev..

[18] Peters C.J., Simpson G.L., Levy H. (1999). Spectrum of Hantavirus Infection: Hemorrhagic Fever with Renal Syndrome and Hantavirus Pulmonary Syndrome. Annu. Rev. Med..

[19] CDC Hemorrhagic Fever with Renal Syndrome (HFRS)—Hantavirus. https://www.cdc.gov/hantavirus/hfrs/index.html#print.

[20] Khan A.S., Khabbaz R.F., Armstrong L.R., Holman R.C., Bauer S.P., Graber J., Strine T., Miller G., Reef S., Tappero J. (1996). Hantavirus Pulmonary Syndrome: The First 100 US Cases. J. Infect. Dis..

[21] CDC Hantavirus Pulmonary Syndrome (HPS)—Hantavirus. https://www.cdc.gov/hantavirus/hps/index.html.

[22] Fulhorst C.F., Milazzo M.L., Armstrong L.R., Childs J.E., Rollin P.E., Khabbaz R., Peters C.J., Ksiazek T.G. (2007). Hantavirus and Arenavirus Antibodies in Persons with Occupational Rodent Exposure, North America. Emerg. Infect. Dis..

[23] Sudi L.E. (2019). Molecular Epidemiology of Rodent-, Shrew-and Bat-Borne Hantaviruses in Mbeya Region, Tanzania. Master’s Thesis.

[24] Martinez V.P., Bellomo C., San Juan J., Pinna D., Forlenza R., Elder M., Padula P.J. (2005). Person-to-Person Transmission of Andes Virus. Emerg. Infect. Dis..

[25] Padula P.J., Edelstein A., Miguel S.D.L., López N.M., Rossi C.M., Rabinovich R.D. (1998). Hantavirus Pulmonary Syndrome Outbreak in Argentina: Molecular Evidence for Person-to-Person Transmission of Andes Virus. Virology.

[26] Martinez-Valdebenito C., Calvo M., Vial C., Mansilla R., Marco C., Palma R.E., Vial P.A., Valdivieso F., Mertz G., Ferrés M. (2014). Person-to-Person Household and Nosocomial Transmission of Andes Hantavirus, Southern Chile, 2011. Emerg. Infect. Dis..

[27] Toledo J., Haby M.M., Reveiz L., Sosa Leon L., Angerami R., Aldighieri S. (2021). Evidence for Human-to-Human Transmission of Hantavirus: A Systematic Review. J. Infect. Dis..

[28] Pini N., Levis S., Calderón G., Ramirez J., Bravo D., Lozano E., Ripoll C., Jeor S.S., Ksiazek T.G., Barquez R.M. (2003). Hantavirus Infection in Humans and Rodents, Northwestern Argentina. Emerg. Infect. Dis..

[29] Castillo C., Villagra E., Sanhueza L., Ferres M., Mardones J., Mertz G.J. (2004). Prevalence of Antibodies to Hantavirus among Family and Health Care Worker Contacts of Persons with Hantavirus Cardiopulmonary Syndrome: Lack of Evidence for Nosocomial Transmission of Andes Virus to Health Care Workers in Chile. Am. J. Trop. Med. Hyg..

[30] Chaparro J., Vega J., Terry W., Vera J.L., Barra B., Meyer R., Peters C.J., Khan A.S., Ksiazek T.G. (1998). Assessment of person-to-person transmission of hantavirus pulmonary syndrome in a Chilean hospital setting. J. Hosp. Infect..

[31] Schountz T., Prescott J. (2014). Hantavirus Immunology of Rodent Reservoirs: Current Status and Future Directions. Viruses.

[32] Wesley C.L., Allen L.J.S., Langlais M. (2010). Models for the Spread and Persistence of Hantavirus Infection in Rodents with Direct and Indirect Transmission. Math. Biosci. Eng..

[33] Guterres A., de Lemos E.R.S. (2018). Hantaviruses and a Neglected Environmental Determinant. One Health.

[34] Song J.W., Gu S.H., Bennett S.N., Arai S., Puorger M., Hilbe M., Yanagihara R. (2007). Seewis Virus, a Genetically Distinct Hantavirus in the Eurasian Common Shrew (*Sorex araneus*). Virol. J..

[35] Gu S.H., Markowski J., Kang H.J., Hejduk J., Sikorska B., Liberski P.P., Yanagihara R. (2013). Boginia Virus, a Newfound Hantavirus Harbored by the Eurasian Water Shrew (*Neomys fodiens*) in Poland. Virol. J..

[36] Klempa B., Fichet-Calvet E., Lecompte E., Auste B., Aniskin V., Meisel H., Barrière P., Koivogui L., Ter Meulen J., Krüger D.H. (2007). Novel Hantavirus Sequences in Shrew, Guinea. Emerg. Infect. Dis..

[37] Kang H.J., Stanley W.T., Esselstyn J.A., Gu S.H., Yanagihara R. (2014). Expanded Host Diversity and Geographic Distribution of Hantaviruses in Sub-Saharan Africa. J. Virol..

[38] Klempa B., Koivogui L., Sylla O., Koulemou K., Auste B., Krüger D.H., Meulen J. (2010). Ter Serological Evidence of Human Hantavirus Infections in Guinea, West Africa. J. Infect. Dis..

[39] Těšíková J., Bryjová A., Bryja J., Lavrenchenko L.A., Goüy De Bellocq J. (2017). Hantavirus Strains in East Africa Related to Western African Hantaviruses. Vector-Borne Zoonotic Dis..

[40] Ogola J.G., Alburkat H., Masika M., Korhonen E., Uusitalo R., Nyaga P., Anzala O., Vapalahti O., Sironen T., Forbes K.M. (2021). Seroevidence of Zoonotic Viruses in Rodents and Humans in Kibera Informal Settlement, Nairobi, Kenya. Vector-Borne Zoonotic Dis..

[41] Omoga D.C.A., Tchouassi D.P., Venter M., Ogola E.O., Eibner G.J., Kopp A., Slothouwer I., Torto B., Junglen S., Sang R. (2022). Circulation of Ngari Virus in Livestock, Kenya. mSphere.

[42] Welcome to the QGIS Project!. https://www.qgis.org/en/site/.

[43] Musila S., Monadjem A., Webala P.W., Patterson B.D., Hutterer R., De Jong Y.A., Butynski T.M., Mwangi G., Chen Z.-Z., Jiang X.-L. (2019). An Annotated Checklist of Mammals of Kenya. Zool. Res..

[44] Kingdon J. (2015). Field Guide to African Mammals.

[45] Langat S.K., Eyase F., Bulimo W., Lutomiah J., Oyola S.O., Imbuga M., Sang R. (2021). Profiling of RNA Viruses in Biting Midges (*Ceratopogonidae*) and Related Diptera from Kenya Using Metagenomics and Metabarcoding Analysis. mSphere.

[46] Marklewitz M., Tchouassi D.P., Hieke C., Heyde V., Torto B., Sang R., Junglen S. (2020). Insights into the Evolutionary Origin of Mediterranean Sandfly Fever Viruses. mSphere.

[47] Clark K., Karsch-Mizrachi I., Lipman D.J., Ostell J., Sayers E.W. (2016). GenBank. Nucleic Acids Res..

[48] Sayers E.W., Bolton E.E., Brister J.R., Canese K., Chan J., Comeau D.C., Connor R., Funk K., Kelly C., Kim S. (2022). Database Resources of the National Center for Biotechnology Information. Nucleic Acids Res..

[49] Altschul S.F., Gish W., Miller W., Myers E.W., Lipman D.J. (1990). Basic Local Alignment Search Tool. J. Mol. Biol..

[50] Edgar R.C. (2004). MUSCLE: Multiple Sequence Alignment with High Accuracy and High Throughput. Nucleic Acids Res..

[51] Guindon S., Dufayard J.F., Lefort V., Anisimova M., Hordijk W., Gascuel O. (2010). New Algorithms and Methods to Estimate Maximum-Likelihood Phylogenies: Assessing the Performance of PhyML 3.0. Syst. Biol..

[52] Phuentshok Y., Dorji K., Zangpo T., Davidson S.A., Takhampunya R., Tenzinla T., Dorjee C., Morris R.S., Jolly P.D., Dorjee S. (2018). Survey and Phylogenetic Analysis of Rodents and Important Rodent-Borne Zoonotic Pathogens in Gedu, Bhutan. Korean J. Parasitol..

[53] Herbreteau V., Jittapalapong S., Rerkamnuaychoke W., Chaval Y., Cosson J.F., Morand S. (2011). Protocols for Field and Laboratory Rodent Studies. http://www.ceropath.org/FichiersComplementaires/Herbreteau_Rodents_protocols_2011.pdf.

[54] Robins J.H., McLenachan P.A., Phillips M.J., McComish B.J., Matisoo-Smith E., Ross H.A. (2010). Evolutionary Relationships and Divergence Times among the Native Rats of Australia. BMC Evol. Biol..

[55] Liyai R., Kimita G., Masakhwe C., Abuom D., Mutai B., Onyango D.M., Waitumbi J. (2021). The Spleen Bacteriome of Wild Rodents and Shrews from Marigat, Baringo County, Kenya. PeerJ.

[56] Heinemann P., Tia M., Alabi A., Anon J.C., Auste B., Essbauer S., Gnionsahe A., Kigninlman H., Klempa B., Kraef C. (2016). Human Infections by Non-Rodent-Associated Hantaviruses in Africa. J. Infect. Dis..

[57] Rodier G., Soliman A., Bouloumie J., Kremer D. (1993). Presence of Antibodies to Hantavirus in Rat and Human Populations of Djibouti.

[58] Witkowski P.T., Leendertz S.A.J., Auste B., Akoua-Koffi C., Schubert G., Klempa B., Muyembe-Tamfum J.J., Karhemere S., Leendertz F.H., Krüger D.H. (2015). Human Seroprevalence Indicating Hantavirus Infections in Tropical Rainforests of Côte d’Ivoire and Democratic Republic of Congo. Front. Microbiol..

[59] Arai S., Bennett S.N., Sumibcay L., Cook J.A., Song J.W., Hope A., Parmenter C., Nerurkar V.R., Yates T.L., Yanagihara R. (2008). Short Report: Phylogenetically Distinct Hantaviruses in the Masked Shrew (*Sorex cinereus*) and Dusky Shrew (*Sorex monticolus*) in the United States. Am. J. Trop. Med. Hyg..

[60] Arai S., Ohdachi S.D., Asakawa M., Hae J.K., Mocz G., Arikawa J., Okabe N., Yanagihara R. (2008). Molecular Phylogeny of a Newfound Hantavirus in the Japanese Shrew Mole (*Urotrichus talpoides*). Proc. Natl. Acad. Sci. USA.

[61] Animalia.bio Somali Shrew—Facts, Diet, Habitat & Pictures. https://animalia.bio/somali-shrew.

[62] Crocidura somalica (Somali Shrew). https://www.iucnredlist.org/species/41359/115181668#habitat-ecology.

